# Hinokitiol inhibits *Aspergillus fumigatus* by interfering with the cell membrane and cell wall

**DOI:** 10.3389/fmicb.2023.1132042

**Published:** 2023-04-11

**Authors:** Fanyue Meng, Xing Liu, Cui Li, Xudong Peng, Qian Wang, Qiang Xu, Jialin Sui, Guiqiu Zhao, Jing Lin

**Affiliations:** Department of Ophthalmology, The Affiliated Hospital of Qingdao University, Qingdao, China

**Keywords:** hinokitiol, *Aspergillus fumigatus*, antifungal, cell wall, cell membrane

## Abstract

Hinokitiol (β-thujaplicin) is an important component of the essential oil extracted from *Chamaecyparis obtuse*, which prevents the decay and decomposition of temple and shrine buildings in Japan. Hinokiol has been shown to have a detrimental effect on various fungi such as *Candida albicans* and *saprophytic* fungi. However how hinokitiol works against *Aspergillus fumigatus* (*A. fumigatus*) has not been claimed. This study aims to investigate the adverse effects of hinokitiol on the disruption of the cell wall and cell membrane of *A. fumigatus* and to explore possible potential mechanisms or pathways. According to our results, hinokitiol negatively altered mycelium morphology, growth density, and cell plasma composition content. When incubated with human corneal epithelial cells (HCECs), hinokitiol saw a safe effect with concentrations below 12 μg/ml. Hinokitiol was shown to increase the cell membrane’s permeability by decreasing the cell membrane’s ergosterol content. The integrity of the cell wall was disrupted, as well as a significant increase in chitin degradation and chitinase activity. As determined by RNA-seq results, subsequent analysis, and qRT-PCR, altered transcript levels of cell walls and cell membranes-related genes (such as eglC) illustrated how hinokitiol affected the genetic profile of *A. fumigatus.* With this study, we recommend hinokitiol as an effective anti-*A. fumigatus* agent by reducing the amounts of key components in the cell wall and membrane by preventing production and accelerating breakdown.

## Introduction

*Aspergillus fumigatus* is considered a potent opportunistic pathogen that can cause a wide range of diseases in humans worldwide (Latgé and Chamilos, 2019). Its ability to adapt and proliferate in harsh environments contributes to resistance and survival against human host defense systems (Latgé and Chamilos, 2019; Fisher et al., 2022). Currently, Aspergillosis can be treated with a few therapeutic agents from three main classes: echinocandins, antimycotics, and amphotericin B (Pasqualotto and Denning, 2008; Wiederhold, 2018; Latgé and Chamilos, 2019). Their antifungal properties are limited by their toxicity and have other drawbacks in clinical applications (Fan et al., 2020). A new need for innovative agents to combat *A. fumigatus* emerged as a result.

Compounds with powerful antifungal properties derived from natural plants have recently received much attention. In Japan, *Chamaecyparis obtuse* is an excellent building material in the construction of Buddhist temples and Shinto shrines (Anderson and Gripenberg, 1948; Inamori et al., 2006). Hinokitiol, a naturally occurring substance associated with *Chamaecyparis obtuse*, was found to have excellent antifungal properties (Komaki et al., 2008; Rebia et al., 2019). Hinokitiol was found to prevent the growth of *Candida* species by disrupting the respiratory chain and blocking the signaling of the Renin-angiotensin system (Komaki et al., 2008; Jin et al., 2021). And it also has a desirable anti-biofilm effect on fluconazole-resistant *Candida albicans* (Kim et al., 2017). Moreover, in Japan, hinokitiol is approved for the treatment of oral candidiasis (Sakaguchi, 2017). It has also been shown to prevent postharvest diseases during the storage of fruits or vegetables, such as those caused by *Fusarium* sp (Wang et al., 2020), *Sclerotinia sclerotiorum* (Qiao et al., 2022), and *Alternaria alternata* (Vanitha et al., 2019).

To date, no studies have reported the antifungal effect of hinokitiol on *A. fumigatus*, and the exact mechanism by which hinokitiol is used to injure *A. fumigatus* is not clear. We hypothesize that hinokitiol acts as an antifungal agent by affecting the cell wall and cell membrane of *A. fumigatus*. In this study, we evaluated specific cell wall and membrane damage conditions and growth inhibition induced by hinokitiol and identified and validated Hub genes by bioinformatics analysis. In conclusion, we provide hinokitiol as a potential new anti-*A. fumigatus* agent.

## Materials and methods

### Chemicals

Sigma-Aldrich Inc. (Shanghai, China) provided the hinokitiol powder (CAS 499–44-5), which was then dissolved into dimethyl sulfoxide (DMSO) to a storage concentration of 32 mg/ml and diluted to acceptable working solutions to reach various final concentrations.

### *Aspergillus fumigatus* strain and culture media

The standard *A. fumigatus* strain (CGMCC 3.14869) was purchased from China General Microbiological Culture Collection Center. Mycelium was obtained following the required number of days (mentioned in the required methods of different experiments) of conidia cultivation on the Sabouraud’s agar medium at 28°C. Conidia on the surface of the medium were collected, counted by a hemocytometer, and adjusted to a final concentration of 1 × 10^7^ /ml.

### Hinokitiol minimum inhibitory concentration to *Aspergillus fumigatus* conidia and time-kill assay

MIC was determined using the standard microdilution method developed by the American Clinical and Laboratory Standards Institute (CLSI) (Wootton et al., 2020). Conidia suspension (2 × 10^5^ CFU/ml) (CFU means colony-forming unit) was cultured with hinokitiol in various concentrations (2, 4, 8, 16, and 32 μg/ml) and 0.1% DMSO at 28°C in 96-well plates (100 μl per well). A microplate reader was used to read the optical density (OD) at 620 nm at 12 h, 24 h, 48 h, 72 h, and 96 h.

### Hinokitiol minimum fungicidal concentration to *Aspergillus fumigatus* conidia

Conidia suspension (2 × 10^5^ CFU/ml) (CFU means colony-forming unit) was cultured with hinokitiol in various concentrations (2, 4, 8, 16, 32,64, and 128 μg/ml) and 0.1% DMSO at 28°C in 96-well plates (100 μl per well). 5 μl of medium from each well was removed and incubated on Sabouraud‘s agar medium for 48 h. When the quantity of CFU (colony forming units) is less than 10, 99% of *A. fumigatus* conidia are killed, and the medication concentration is the minimal fungicidal concentration.

### Hinokitiol MIC to *Aspergillus fumigatus* mycelium

Conidia suspension (2 × 10^5^ CFU/ml) was cultured for 24 h at 28°C in 96-well plates (100 μl per well) to generate mycelium. 2, 4, 8, 16, and 32 μg/ml of hinokitiol were added with 0.1% DMSO to each well before being incubated for 24 h. A microplate reader was used to read the OD at 620 nm.

### The mammalian cell culture and viability assay

HCECs (provided by University of Xiamen, Fujian, China) were incubated according to the previous method by Yin et al. (2022). Cell suspension (3 × 10^5^/mL) to be tested was incubated in a 96-well plate with different concentrations of hinokitiol for 24 h under the original condition in dark. The absorbance values were measured at 450 nm 3 h after the addition of 10 μl of cell counting kit-8 (CCK-8) each well. 6 replicates were set up for each sample.

### Transmission electron microscope

We seeded the *A. fumigatus* conidia (2 × 10^5^ CFU/ml) into 6-well plates and cultured there for 24 h at 28°C to generate mycelium. After being washed the mycelium was centrifuged at 12,000 rpm for 10 min before being transferred to a fresh 6-well plate. The mycelium was rinsed with phosphate buffer (PBS), then collected and fixed with 2.5% glutaraldehyde at 4°C overnight after being incubated with 0.1% DMSO or hinokitiol (8 μg/ml) at 28°C for 24 h. The following procedures were followed as per standard procedures to prepare the mycelium sample for TEM observation (Shen et al., 2020). We examined the samples using a JEOL-1200EX transmission electron microscope.

### Scanning electron microscope

We seeded the *A. fumigatus* conidia (2 × 10^5^ CFU/ml) into 6-well plates and cultured there for 24 h at 28°C to generate mycelium. The mycelium was washed, centrifuged at 12,000 rpm for 10 min, and then transferred to a new 6-well plate. The mycelium was rinsed with PBS after being incubated with 0.1% DMSO or hinokitiol (8 μg/ml) at 28°C for 24 h. They were then collected and fixed with 2.5% glutaraldehyde at 4°C for 2 h. PBS was used to clean the samples, and they were then mixed for an hour at 4°C with 1% (v/v) osmium tetroxide in PBS. Samples were then gently dehydrated in graded ethanol, critical point dried in CO2, coated with gold, and seen under an SEM (VEGA3; TESCAN Company, Shanghai, China) at magnifications of 2000 and 5,000 (bar =20 or 10 μm).

### Cell membrane permeability assay

*Aspergillus fumigatus* mycelium was prepared and collected as previously indicated and was treated with hinokitiol (4 μg/ml, 8 μg/ml) or without hinokitiol (0.1% DMSO) for 24 h, 36 h, or 48 h. Each group received a 15-min addition of 50 μg/ml propidium iodide (PI) solution before being incubated in the dark at room temperature. Bright-field and fluorescence images were both taken using a fluorescence microscope (Nikon, Tokyo, Japan, 100×) with green excitation light or not. Image J software was used to calculate the fluorescence intensity.

### Ergosterol content determination

*Aspergillus fumigatus* mycelium was prepared in sabouraud medium for 5 days and was treated with hinokitiol (4 μg/ml, 8 μg/ml) or without hinokitiol (0.1% DMSO) for 48 h. Mycelium was washed in sterile distilled water, filtered, and fully dried. Total ergosterol contents of *A. fumigatus* mycelium with different hinokitiol treatments were determined by the method described previously (OuYang et al., 2019) with a slight modification. 48 h were spent vacuum-freezing the samples before 0.1 g of each group was saponified in 4 ml of freshly made 30% (w/v) methanolic KOH and 8 ml of 100% ethanol at 90°C. 3 ml of petroleum ether was used three times to extract the mixtures containing 10 ml of saturated sodium chloride solution. After placing the samples in the extraction flask, the supernatants were collected. At 60 to 90°C, the petroleum ether solution was then distilled. With the help of 10 ml anhydrous ethanol, the solute crystallized and was retrieved. High-Performance Liquid Chromatography (HPLC) analysis was carried out using a HITACHI high-performance liquid chromatograph (Chromaster, HITA-CHI, Japan). At a constant temperature, the sample extracts were separated and examined on a C18 column (250 mm × 4.6 mm, 5 μm). The mobile phase was made up of Solvent A (methanol). A flow of 1 ml per minute was present. Chromatographic peaks were discovered by measuring retention times and spectra against the specified standard. The detection wavelength was set at 282 nm. For the analysis, 20 μl aliquots were directly injected into the HPLC. Ergosterol standard curve is provided in Supplementary Table S1.

### Cell wall integrity assay

*Aspergillus fumigatus* mycelium was prepared and collected as previously indicated and the experimental groups and the control groups were classified using the same approach as in the cell membrane permeability assay. We utilized calcofluor white (Sigma, St. Louis, MO, United States) and fluorescence microscopy to observe. The collected mycelia were stained with 10 μl KOH (10%) followed by 10 μl of calcofluor white stain as the manufacturer’s instructions.

### Chitin content determination

The experimental groups and the control groups were classified using the same approach as in the cell membrane permeability assay. We assayed chitin content according to the previous method described with slight modifications (Ke et al., 2022). The mycelium samples were cultured in a 96-well plate. They were twice rinsed with distilled water before being incubated for 5 min in the dark with Congo red (0.025%). The materials were examined using a microplate reader (Tecan, Switzerland) with excitation and emission wavelengths of 540 and 600–850 nm, respectively.

### Chitinase activity determination

*Aspergillus fumigatus* mycelium was prepared in sabouraud medium for 5 days and was treated with hinokitiol (4 μg/ml, 8 μg/ml) or without hinokitiol (0.1% DMSO) for 24 and 48 h. Mycelium was washed in sterile distilled water, filtered, and fully dried. We ground the mycelium into a paste with the chitinase extraction reagent in an ice bath and then centrifuged them at 8000 rpm at 4°C for 25 min. The supernatant was collected to get the extracted enzyme solution, and a commercial BC0820 kit was used to measure chitinase activity (Solarbio Technology, Beijing, China).

### RNA-seq and cytoscape analysis

Conidia suspension (2 × 10^5^/mL) containing 0.1% DMSO or hinokitiol (4 μg/ml) was incubated on a 6-well plate at 28°C. The Control (DMSO treated) and Test (HK treated) samples were harvested after incubation for 36 h and then stored at −80°C for RNA-Seq analysis. All the experiments were carried out in 3 independent biological replicates. Total RNA was extracted using the TRIzol reagent according to the manufacturer’s protocol. RNA purity and quantification were evaluated using the NanoDrop 2000 spectrophotometer (Thermo Scientific, United States). RNA integrity was assessed using the Agilent 2,100 Bioanalyzer (Agilent Technologies, Santa Clara, CA, United States). Then the libraries were constructed using TruSeq Stranded mRNA LT Sample Prep Kit (Illumina, San Diego, CA, USA) according to the as manufacturer’s instructions. The transcriptome sequencing and analysis were conducted by OE Biotech Co., Ltd. (Shanghai, China). The Sequence Read Archive has been released in NCBI^1^ and the 6 runs can be searched by accession numbers SRR22582946, SRR22582945, SRR22582944, SRR22582943, SRR22582942, and SRR22582941.

Hub genes were screened from the differently expressed genes (DEGs) in the RNA-seq result. We utilized the STRING database (version 11.5^2^) to analyze the protein–protein interactions (PPI) of Hub DEGs. Cytoscape software (version 3.8.0) was used to visualize the PPI network. The Molecular Complex Detection (MCODE) plugin of Cytoscape was then used to determine the important gene modules in the PPI networks. Finally, the cytoHubba plugin was used to rank the genes and highlight Hub genes. Overall, the features we got from different plugins were represented in the PPI network.

### Quantitative real-time polymerase chain reaction

Conidia suspension (2 × 10^5^/mL) containing 0.1% DMSO or hinokitiol (4 μg/ml) was incubated on a 6-well plate at 28°C. The Control (DMSO treated) and Test (HK treated) samples were harvested after incubation for 36 h. The total RNA was prepared using the Fungal Total RNA198 Isolation Kit (B518629, Sangon Biotech, Shanghai, China), and reverse transcribed with HiScript III RT SuperMix (Vazyme, Nanjing, China) according to the manufacturer’s instructions. The PCR method was based on previous studies (Sun et al., 2019). Primers used for the qRT-PCR are listed in Supplementary Table S2.

### Statistical analysis

For comparisons with data from the control group or HK treatment group, we performed an unpaired, two-tailed Student’s t-test or one-way ANOVA with *post hoc* analysis. GraphPadPrism 8.0 (San Diego, California, United States) and ImageJ1.44p (National Institutes of Mental Health, USA) were used for statistical analyzes, and values were presented as the mean ± standard deviation (SD). *p* < 0.05 (*p < 0.05, **p < 0.01, ***p < 0.001) was considered statistically significant (ns = no significance). All the experiments were carried out in 3 independent biological replicates.

## Results

### Hinokitiol damaged the growth of *Aspergillus fumigatus*

**Figure 1:**
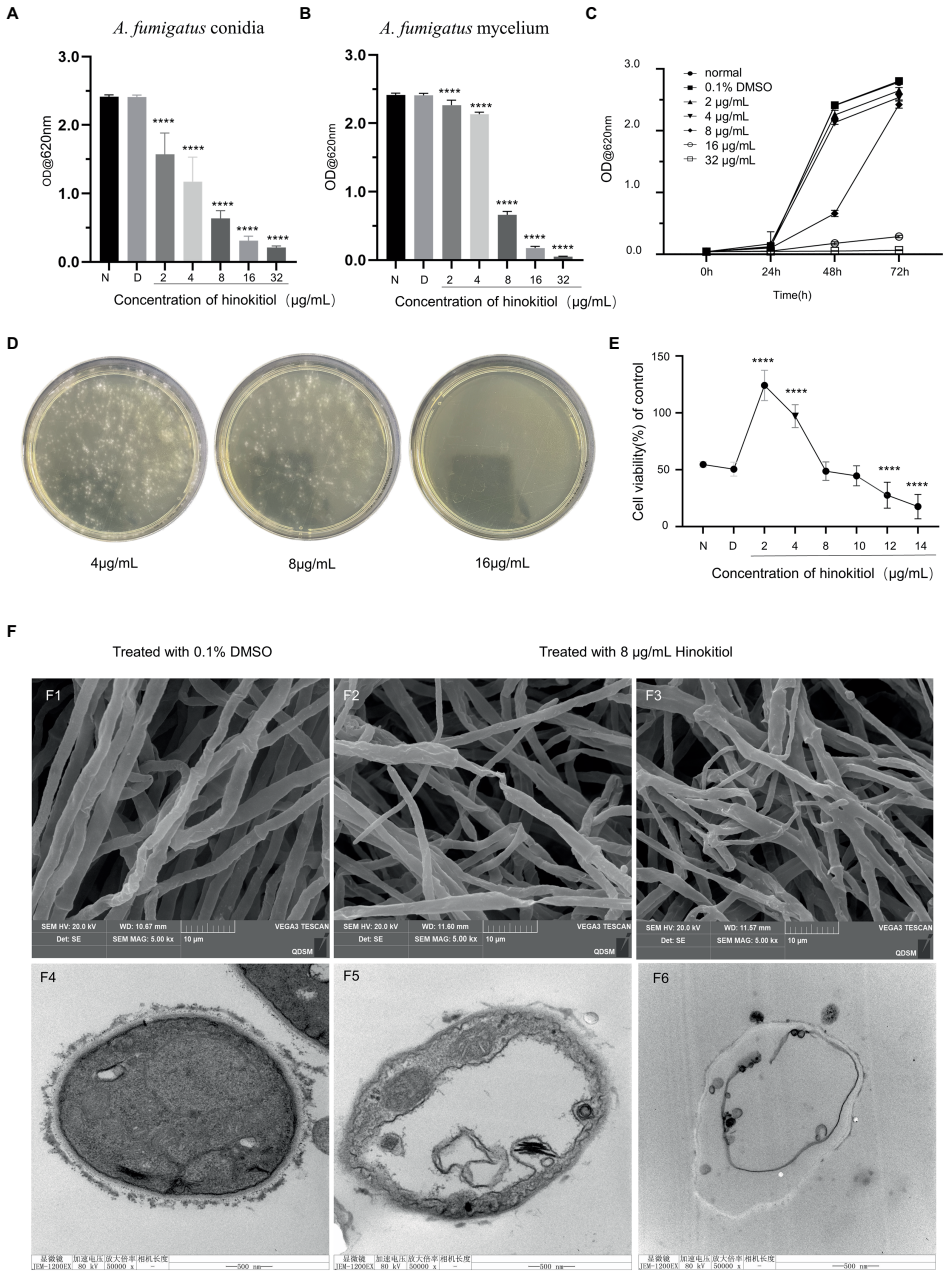
Antifungal activity of hinokitiol against *A. fumigatus*. **(A)** *A. fumigatus* conidia MIC. **(B)** *A. fumigatus* mycelium MIC. N means the normal group and D means 0.1% DMSO group. **(C)** Time-kill curve. **(D)** MFC assay. **(E)** HCECs viability assay. **(F)** F1-3 are SEM and F4–6 are TEM. F1,4 are untreated control groups, F2-3 and F5-6 are treated with 8 μg/ml hinokitiol (**** *p* < 0.0001).

According to MIC data, the germination of *A. fumigatus* conidia and growth of *A. fumigatus* mycelium were inhibited by hinokitiol at 2 μg/ml, with the extent varying proportionally to concentrations from 2 to 32 μg/ml (Figures 1A,B). The 0.1% DMSO treatment group showed no meaningful effect on the growth of *A. fumigatus* compared to the control group. The time-kill curve in Figure 1C showed that as the hinokitiol dose increased, the absorbance values at the same time point gradually decreased, with a higher difference at 48 h between 4 μg/ml and 8 μg/ml treated groups. MFC assay results indicated that *A. fumigatus* conidia were 100% killed by hinokitiol at 16 μg/ml (Figure 1D).

### The cytotoxicity evaluation of hinokitiol on the mammalian cell

Fungal keratitis is a serious blinding eye disease caused by a fungal infection, which has an acute onset, rapid progression, and poor prognosis. The common causative fungi are *Aspergillus* spp., *Candida* spp. and *Fusarium* spp. (Brown et al., 2021; Sharma et al., 2022).The effect of hinokitiol on the proliferative capacity of HCECs was investigated, and the results are shown in Figure 1E. The proliferative capacity of HCECs experienced a significant rise at 2 and 4 μg/ml of hinokitiol, and then saw a gradual drop thereafter. With the exposure of 8 and 10 μg/ml of hinokitiol, no differences in cell proliferation degree were seen compared to the control group. It was not until 12 μg/ml of hinokitiol that HCECs were inhibited to grow. Therefore, hibokitiol with concentrations being below 10 μg/ml is considered to be high-safe and low-toxic.

### Hinokitiol affected the structure and morphology of *Aspergillus fumigatus*

We used TEM and SEM to examine the micromorphological alterations in hinokitiol-treated *A. fumigatus*, focusing on the cell wall and cell membrane. SEM revealed that hinokitiol-treated mycelium grew in a chaotic and uneven manner (Figures 1F2,F3) compared to the control (Figure 1F1). They atrophied and deformed, collapsed or flattened and hollowed. The mycelium in Figure 1F2 had twisted and virtually shattered shapes. Figure 1F3 also showed branching and odd shape. TEM results of mycelium ultrastructure displayed that the cell membrane was coiled, discontinuous, and inconspicuous under the action of hinokitiol. The cell membrane separated from the cell wall became ruptured (Figures 1F5,F6). The hinokitiol-treated group likewise exhibited a fuzzy structure of the cell wall that dissolved into fragments and was noticeably shallower than in the control group (Figures 1F5,F6). The findings suggested that hinokitiol interfered with cell wall integrity and cell membrane continuity in some way.

### Hinokitiol increased the cell membrane permeability

**Figure 2:**
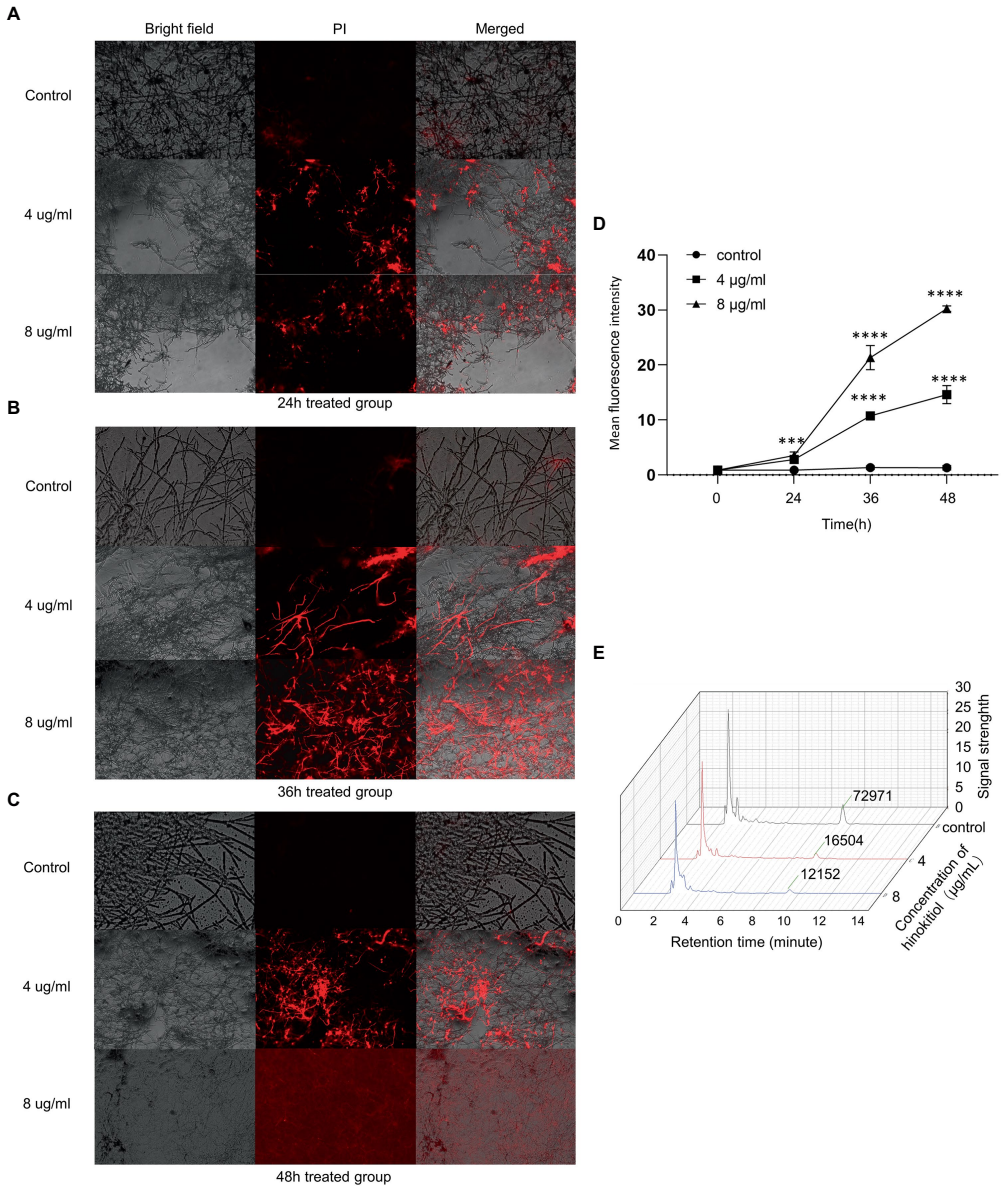
The cell membrane was influenced by hinokitiol. **(A–C)** PI staining. Bright-field and fluorescence images were taken with a fluorescence microscope. The control group was treated with 0.1% DMSO. **(D)** ImageJ analysis. **(E)** HPLC. The extracted ingredient retrieved by ethanol was examined by a high-performance liquid chromatograph. The numbers at around 10 min mean the levels of ergosterol content. The control group was treated with 0.1% DMSO (*** *p* < 0.001, **** *p* < 0.0001).

We used a propidium iodide dye that is only taken up by cells with impaired cell integrity to evaluate cell membrane permeability. Hinokitiol (4 g/ml, 8 g/ml) was applied to the mycelium for 24 h, 36 h, and 48 h. In bright fields, *A. fumigatus* mycelium width and density decreased with medication concentrations and length of time, while the fluorescence intensity in fluorescence images rose gradually (Figures 2A–C). Besides that, the percentage of mycelium stained by PI solution also increased in the same manner. The fluorescence was extensively distributed in the field of vision in the 8 g/ml, 48-h treatment group, and the brilliant spots representing exudation were extremely dense. We utilized the software ImageJ to quantitatively clarified the fluorescence intensity in each PI staining field which showed a clear rising trend over time and across concentration (Figure 2D). Overall, hinokitiol was shown to have a detrimental impact on the pattern of mycelium growth, cell integrity; and increased cell membrane permeability depending on the duration of action and drug concentration proporationally.

### Hinokitiol decreased cell membrane component

Quantitative measurement of the main component of *A. fumigatus* cell membrane, ergosterol, was assayed and the ratio of dry weight (DW) of mycelium was calculated. We demonstrated that there was a substantial difference in ergosterol levels between each group with or without hinokitiol treatment. After being exposed to hinokitiol with 4 or 8 μg/ml for 48 h, the HPLC result showed that ergosterol content decreased compared with the control group (Figure 2E). The ratios of DW in the control, 4 μg/ml, 8 μg/ml group were 5.160734 mg/g, 0.664238 mg/g, and 0.317686 mg/g, respectively. Therefore, we found that hinokitiol may compromise cell membrane integrity by losing ergosterol content in cell membranes.

### Hinokitiol affected cell wall integrity

**Figure 3:**
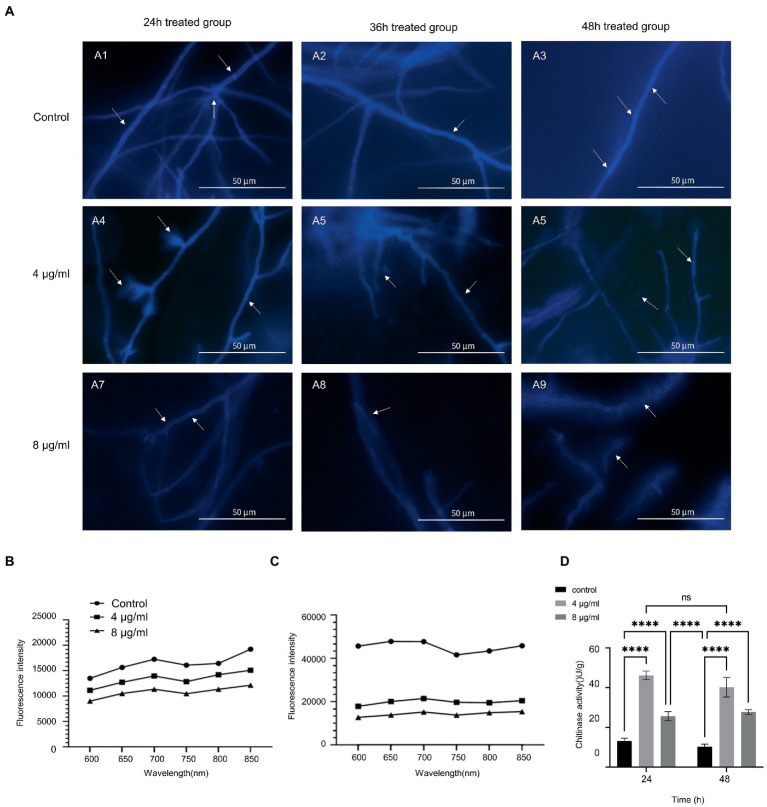
Hinokitiol targeted the cell wall. **(A)** Calcofluor white staining. Fluorescence images were taken with a fluorescence microscope. **(B,C)** Congo red staining. Microplate reader with excitation and emission wavelengths of 540 and 600–850 nm, respectively. **(D)** Chitinase activity. (*n* = 3/group; ns *P* > 0.05, **** *p* < 0.0001).

We tested calcofluor white staining to explore how hinokitol damage *A. fumigatus* cell wall. Control mycelium witnessed a strong fluorescence intensity in its chitin-rich cell wall and septa (Figures 3A1–A3). After being treated with hinokitiol, the overall fluorescence intensity of the cell wall and septum fell in a hinokitiol concentration and time-dependent manner (Figures 3A4–A9). In addition, we found that in the 8 μg/ml treatment group especially, the fluorescence profile became blurred and the background fluorescence was greatly increased as indicated by arrows (Figures 3A7–A9). It suggested that the cell wall was disrupted by hinokitiol, where the chitin content leaked out and dispersed. Besides that, the fungus cultured with hinokitiol became narrower in diameter, more branched, and shorter in mycelium length.

### Hinokitiol reduced chitin contents in cell walls

Then the chitin content of *A. fumigatus* in the control and treatment groups was further measured (Figures 3B,C). Both graphs displayed that hinokitiol reduced the chitin content compared to the group without hinokitiol. In the comparison between the different exposure period groups, after being treated with hinokitiol, the increase degrees in chitin content levels were smaller in the 4 and 8 μg/ml hinokitiol-treated groups than that in the control group, for the difference in chitin content levels between the control and treated groups increased in the same time point. The results indicated that hinokitiol disrupted the cell wall components, and chitin, and inhibited the synthesis of cell wall components.

### Hinokitiol influenced chitinase activity

Subsequently, we examined the activity of chitinase in *A. fumigatus* after treatment with different concentrations of hinokitiol for 24 and 48 h (Figure 3D). Overall, chitinase activity proved to increase in hinokitiol-treated groups in both 24 h and 48 h group (*p* < 0.0001), despite seeing a drop in chitinase activity at 8 μg/ml (*p*<0.0001). With the same concentration of hinokitol, there was no statistically significant difference between the chitinase activities of the two groups at two treatment periods (*p*>0.05). The figure suggested that hinokitiol influenced the biological activity of chitinase, which might explain why the chitin content of the *A. fumigatus* cell wall decreased after hinokitiol exposure. And the higher concentration of hinokitiol could be able to damage the whole bioactivity, responsible for the fall of chitinase activity at 8 μg/ml.

### RNA-seq analysis and validation

**Figure 4:**
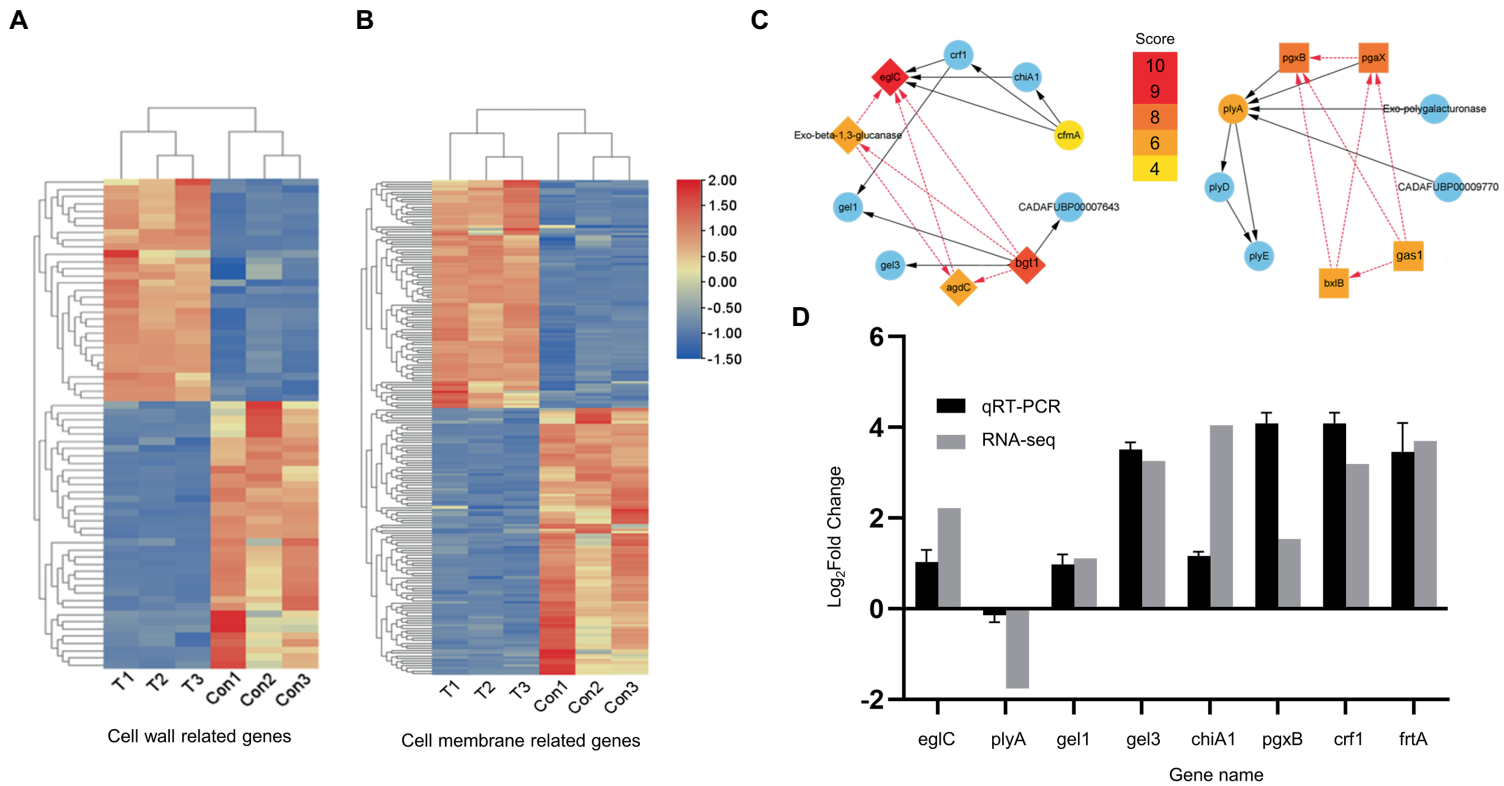
RNA-seq analysis and qRT-RNA validation. **(A,B)** Heat maps. Related to the cell wall and cell membrane, respectively. **(C)** PPI network. Gene screening and STRING database analysis were performed on Hub genes in advance. **(D)** qRT-PCR validation. The up direction of bars represents increased expressions of genes and the down direction of bars means decreased expressions of genes.

To explore the effects at the gene level, we used RNA-seq analysis to investigate the transcriptional characteristics of *A. fumigatus* treated with (4 μg/ml, 36 h) or without hinokitiol treatment. RNA-seq results and derived DEGs related to the cell wall or cell membrane remodeling were subsequently analyzed and divided into two heat maps (Figures 4A,B). There are 67 genes in Figure 4A and 197 genes in Figure 4B. The heat maps provide a global view of specific DEGs, with red representing up-regulation trends and blue representing down-regulation trends. Figure 4A displays genes associated with the fungal cell wall, most of which are involved in the synthesis and degradation of mannans, glucan, and chitin, in addition to some genes related to the activities of the fungal cell wall such as germination, septogenesis, and extracellular sensing. Figure 4B shows genes related to fungal cell membranes, which are involved in the transmembrane transport of various substances in fungi, the formation of cell membranes, regulation of ion channel activity, and mediating the membrane structure with the endoplasmic reticulum, Golgi apparatus, and other organelles to form an integral membrane structure.

Nineteen hub genes associated with the cell wall (Figure 4C) and 10 hub genes related to the cell membrane (Supplementary Figure S3) were obtained and were considered to play an important function in *A. fumigatus* cultured with hinokitiol (Figure 4C). *EglC*, *pgxB*, *plyA*, *gel3*, *gel1*, *chiA1*, *crf1*, and *ftrA* from the PPI network performed qRT-PCR experiments. Among them, *eglC*, which encodes an endoglucanase, is thought to play the most indispensable role. PCR results validated the reliability of RNA-seq, as the expression trends of these genes were the same with those of heat maps, albeit to a different extent (Figure 4D).

## Discussion

Hinokitiol, also named β-thujaplicin, was shown to be biologically active against harmful insects and wood decay fungi because of its protection of wooden structures in Japan for almost 800 years (INAMORI et al., 2000). It is reported to be extracted from the acid oil produced by distillation of the building material *Chamaecyparis obtuse* and has the same basic structural elements as tropolone, which is known for its ability to repel termites. Research interests have been greatly stimulated by the antifungal properties of hinokitiol. As a powerful antifungal agent and potential defense against *Candida albicans*, hinokitiol has been demonstrated by previous studies. In our study, we verified the presence of an impaired state by measuring the absorbance of conidia and mycelium growth of *A. fumigatus* as well as micromorphological findings by electron microscopy. In addition, HCECs proliferative capacity was not significantly inhibited by hinokitiol (under 12 μg/ml), indicating hinokitiol as a potential agent with potential for application for humans. Overall, hinokitiol is likely to be a promising drug for the treatment of fungal infectious diseases.

Subsequently, we found that the cell membrane was significantly disrupted by the disturbance of hinokitiol by various experimental methods; with the increase of treatment dose and time, cell membrane permeability increased and the amount of ergosterol in the cell membrane decreased. The integrity of the cell membrane was critical in preserving fungal vitality (OuYang et al., 2019). As a promising drug target, cell membranes have been studied to achieve effective medicinal development (Sant et al., 2016). Their disruption could produce alterations in hyphae due to increased cell permeabilization, which usually means leakage of small molecular compounds and ions, lesions, and inconsistencies in cell metabolism (Shao et al., 2013; Guo et al., 2022). For example, AgNPs can disrupt cell membrane permeability and disrupt lipid bilayers causing ion leakage (Abdallah et al., 2023). The main components of the cell membrane of fungi are various lipids divided into three classes which are glycerophospholipids, sphingolipids, and sterols. The reduction of lipid composition results in a loss of cell membrane structural stability increased permeability to water-soluble materials, and morphological change (Tao et al., 2014; OuYang et al., 2016). Among them, ergosterol is the most abundant sterol component of them and is specific to fungi (Sant et al., 2016). Previous studies suggested that the impairment of ergosterol biosynthesis was the mechanism of damaging cell membranes of fungi of a few valid antifungal drugs (Tian et al., 2012; Guo et al., 2022). Previous study showed that nystatin can bind ergosterol to form broken channels in the cell membrane and alter permeability by causing the outflow of sodium, potassium and hydrogen ions (Thompson et al., 2009). Imidazole and triazoles were considered to be the most successful antifungal drugs targeting ergosterol synthesis (Sant et al., 2016). Aside from providing a protective barrier, cell membrane also plays critical roles in promoting virulence because of the mediation of the secretion of virulence factors, endocytosis, cell wall synthesis, and invasive mycelium morphogenesis (Douglas and Konopka, 2016). Therefore, based on the above results, hinokitiol proved to be detrimental to the cell membrane, and the decrease in ergosterol content may lead to diminished virulence of *A. fumigatus*.

Because of its roles in shielding itself from external distraction, engaging with the host immune system through particular virulence components, and being a potential target for antifungal drugs, the cell wall is thought to be a crucial organelle of *A. fumigatus*, which was also considered to be one of the virulence factors (Bowman and Free, 2006; Latgé, 2007; Dichtl et al., 2012; Latgé and Chamilos, 2019). It is composed of a branched β-1,3-glucan as a core skeleton, to which chitin, β-1,4-glucan, and galactomannan are linked, surrounded by other polysaccharides as a cement, such as α-1,3-glucan and mannans (van de Veerdonk et al., 2017; Latgé and Chamilos, 2019). Chitin is an integral part of the fungal cell wall and, together with β-1,3-glucan, plays a key role in maintaining cellular integrity and can confer structural rigidity during growth and morphogenesis (Corrêa et al., 2020). Chitin accounts for 10–20% of the dry weight of the cell wall of *A. fumigatus* and is of considerable inportance. When *A. fumigatus* is placed in a difficult environment, a cell wall’s dynamic structure can be altered in a variety of ways to withstand its harmful effects (van de Veerdonk et al., 2017). Under normal conditions, the composition and degradation of chitin occur in a dynamic equilibrium, which, once disrupted, is likely to lead to cell death (Ke et al., 2022). For example, in previous studies, drugs such as cinnamaldehyde, propolis extract and chitosan oligosaccharides were able to target chitin as the main target for fungal destructive effects (OuYang et al., 2019; Corrêa et al., 2020; Ke et al., 2022). In our study, we found that hinokitiol was able to cause roughness and unevenness of the cell wall, shortening, fracture and blurring of the mycelium, and reduction, leakage, and dispersion of chitin in the cell wall and septum. The loss of chitin content, interfering biosynthesis, and triggering degradation leading to deformity and broken of *A. fumigatus* were observed in our reports. In addition, chitinases are present in all fungal species and play an important role in building and maintaining the cell wall structure and function of fungi. The breakdown of chitin by chitinase leads to the disruption of cell wall integrity and thus participates in the morphogenesis of the fungus (Roncero and Vázquez de Aldana, 2020). The anti-cell wall activities of hinokitiol may spring from the ability to disrupt the balance of chitin synthesis and decomposition, which results in morphological abnormalities and even fragmentation and fracture of fungal cells, leading to loss of cell wall integrity and osmotic instability.

To further study the antifungal mechanism of hinokitiol on a molecular level, we analyzed RNA-seq data of *A. fumigatus* treated by hinokitiol or not by gene screening, STRING database analysis, PPI protein network analysis, Hub gene selection, and gene function determination. Besides that, we verified the reliability of RNA-seq results by PCR. Hub genes we got explained the possible influence manner by which hinokitiol did to *A. fumigatus*. *EglC*, *bgt1*, *pgxB*, *plyA*, *gel3*, *gel1*, *chiA1*, *crf1*, and *ftrA* were performed by qRT-PCR and the reliability of RNA-seq was validated. *EglC,* encoding an endoglucanase, has been recently suggested its greatest action on polysaccharide (Hasper et al., 2002). The endoglucanase EglC is able to inhibit the growth of *A. fumigatus* by degrading cell walls (Champer et al., 2016) and also engages in the delivery of proteins anchored to the cell membrane in *Aspergillus nidulans*. *EglC* is also involved in the delivery of proteins anchored to the Aspergillus nidulans cell membrane. Previous studies have shown that eglC in *Aspergillus nidulans* is expressed as a GPI-anchored protein, which can be delivered to the cell membrane surface in a polarized manner and is involved in the composition of lipid and ergosterol of cell membrane (Peñalva et al., 2020). In our research, *eglc* was considered as the most indispensable gene related to both cell walls and cell membranes and its upregulated expression in the exposure of hinokitiol may be important for its ability to cause increased xyloglucan hydrolysis (Hasper et al., 2002), disruption of cell wall structure and homeostasis imbalance; it is possible that the upregulated transcript level of *eglC* is due to the negative feedback of reduced GPI after cell membrane disruption. *Gel1* and *gel3*, belonging to the β-1,3-glucanosyltransferase gels family(gel1-7), were essential for both morphogenesis and virulence in *A. fumigatus* for the elongating and branching activities of β-1,3-glucan chains (Mouyna et al., 2005; Zhao et al., 2013, 2014; Aimanianda et al., 2017). *Crf1* from the transglycosylases crfs (crf1-2) family has been reported in articles, which is responsible for cross-linking of chitin and glucan (Arroyo et al., 2007). *Crf1* and *gel1* were considered to be the candidates for a promising vaccine as their highly expressed conserved character in *A. fumigatus* (Arroyo et al., 2007). *Gas* and *crh* family from *Saccharomyces cerevisiae* were mentioned in previous research. Poacic acid is a cell wall-targeted antifungal agent exhibiting its function of inhibiting the activity of gas and crh (Piotrowski et al., 2015; García et al., 2021), which are orthologous protein families of gel and crf from *A. fumigatus*, respectively (Arroyo et al., 2007). The activity of these enzymes plays an indispensable role in constructing the outer skeleton of fungi under pressures outside and are expected to be antifungal drug targets. Our research elaborated that the significantly different expression pattern of *eglC*, *gel1*, *gel3*, and *crf1* compared with the control group and then their critical positions of them in the PPI network has also been highlighted.

Studies have shown that the disruption of the yeast cell wall can initiate the CWI (cell wall integrity) pathway and induce changes in gene transcription profiles (García et al., 2021). Hinokitiol, by interfering with the expression of these enzyme genes, may prevent the elongation and branching of β-1,3-glucan, inhibit the maturation process of glycosylation, and disturb the cross-linkng of glucan and chitin. The expression level of *chiA1*(one of the chitinases family) increased in RNA-seq which probably means chitin hydrolysis was enhanced by hinokitiol in the cell wall of *A. fumigatus*, and probably explained the reason for the elevated chitinase activity in our previous experiment (Yang and Zhang, 2019). *FtrA* encodes high-affinity iron permease, with the ability to modulate iron ion transmembrane transport (Li et al., 2023). Previous studies showed that hinokitiol could disrupt iron homeostasis, leading to respiratory chain dysfunction of *Candida* strains (Jin et al., 2021). Here we found that the interfering process might involve the engagement of cell membranes and Fe transporter permease *ftrA.* Therefore, we demonstrate that the influence of these genes in the involvement of cell wall and cell membrane structure and function is not negligible, which further illustrates the ability of hinokitiol to damage fungi.

Overall, RNA-seq results revealed their expression pattern differences and demonstrated that they may play an important role in participating in cell wall remodeling by affecting the expression of several hydrolytic enzyme. The synthesis, prolongation, maturation, and cross-linking of glycan components were disturbed, thus affecting the formation of the cell wall; in addition, the catabolism of cell wall components may be promoted by up-regulation of hydrolase gene transcription, and the content of polysaccharides and chitin decreased, ultimately leading to the rupture of the cell wall structure. While altered expression of cell membrane-associated genes *eglC* and *ftrA* was also identified to be responsible for the function of hinokitiol; altered expression of fungal extracellular structure-anchoring proteins and transport permeases may have affected cell membrane permeability and stability.

In conclusion, our present studies revealed that hinokitiol altered the morphology of *A. fumigatus*. Hinokitiol interfered with the ergosterol biosynthesis process to disrupt the normal structures of membranes, resulting in component leakage of cells and reducing ergosterol content in cell membranes. Hinokitiol also promoted the hydrolysis process of polysaccharide components, inhibited chitin biosynthesis in cell walls, and caused the malformation of fungi by influencing chitinase activity and the expressions of Hub genes. All the negative alteration of cell integrity eventually led to a bioactivity and virulence reduction of *A. fumigatus* much likely. These results suggested that hinokitiol acted its antifungal function against *A. fumigatus* by targeting the cell membrane and cell wall.

## Data availability statement

The datasets presented in this study can be found in online repositories. The names of the repository/repositories and accession number(s) can be found in the article/Supplementary material.

## Author contributions

FM designed, and implemented most of the research and wrote the first version of the manuscript. XL conducted vital parts of the experiments and reviewed the manuscript. CL reviewed and edited the manuscript. XP, QW, and QX collected and analyzed the data. GZ and JL supervised the study. All authors read and approved the manuscript.

## Funding

This work was supported by Taishan Scholar Project of Shandong Province [grant numbers tsqn201812151].

## Conflict of interest

The authors declare that the research was conducted in the absence of any commercial or financial relationships that could be construed as a potential conflict of interest.

## Publisher’s note

All claims expressed in this article are solely those of the authors and do not necessarily represent those of their affiliated organizations, or those of the publisher, the editors and the reviewers. Any product that may be evaluated in this article, or claim that may be made by its manufacturer, is not guaranteed or endorsed by the publisher.

## Footnotes

^1^ https://www.ncbi.nlm.nih.gov/

^2^ https://string-db.org/
