# Supplementary figures and images for: Hinokitiol inhibits Aspergillus fumigatus by interfering with the cell membrane and cell wall
Source: Front Microbiol. 2023 Apr 11;14:1132042. doi: 10.3389/fmicb.2023.1132042 (PMC10128913; doi:10.3389/fmicb.2023.1132042)

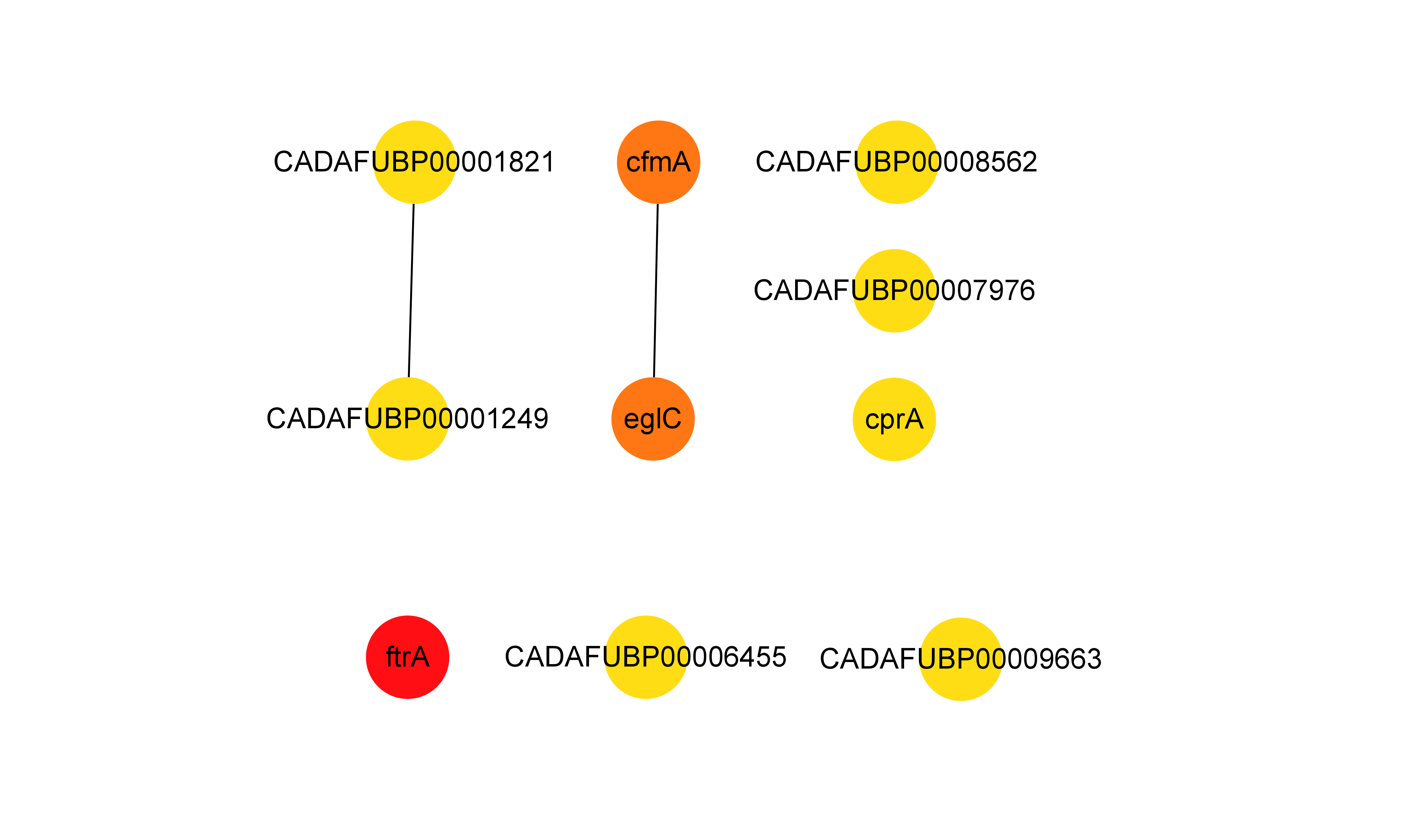

Supplement: Supplementary file 3 [file Image_1.PNG]
